# A Novel Pathway Regulates Thyroid Hormone Availability in Rat and Human Hypothalamic Neurosecretory Neurons

**DOI:** 10.1371/journal.pone.0037860

**Published:** 2012-06-18

**Authors:** Imre Kalló, Petra Mohácsik, Barbara Vida, Anikó Zeöld, Zsuzsanna Bardóczi, Ann Marie Zavacki, Erzsébet Farkas, Andrea Kádár, Erik Hrabovszky, Rafael Arrojo e Drigo, Liping Dong, László Barna, Miklós Palkovits, Beáta A. Borsay, László Herczeg, Ronald M. Lechan, Antonio C. Bianco, Zsolt Liposits, Csaba Fekete, Balázs Gereben

**Affiliations:** 1 Department of Endocrine Neurobiology, Institute of Experimental Medicine, Hungarian Academy of Sciences, Budapest, Hungary; 2 Department of Neuroscience, Faculty of Information Technology, Pázmány Péter Catholic University, Budapest, Hungary; 3 Thyroid Section, Division of Endocrinology, Diabetes and Hypertension, Brigham and Women’s Hospital and Harvard Medical School, Boston, Massachusetts, United States of America; 4 Division of Endocrinology, Diabetes and Metabolism, University of Miami Miller School of Medicine Miami, Florida, United States of America; 5 Nikon Microscopy Center, Institute of Experimental Medicine, Budapest, Hungary; 6 Human Brain Tissue Bank, Semmelweis University, Budapest, Hungary; 7 Department of Forensic Medicine, Faculty of Medicine, University of Debrecen, Debrecen, Hungary; 8 Tupper Research Institute and Department of Medicine, Division of Endocrinology, Diabetes, and Metabolism, Boston, Tufts Medical Center, Boston, Massachusetts, United States of America; University Claude Bernard Lyon 1, France

## Abstract

Hypothalamic neurosecretory systems are fundamental regulatory circuits influenced by thyroid hormone. Monocarboxylate-transporter-8 (MCT8)-mediated uptake of thyroid hormone followed by type 3 deiodinase (D3)-catalyzed inactivation represent limiting regulatory factors of neuronal T3 availability. In the present study we addressed the localization and subcellular distribution of D3 and MCT8 in neurosecretory neurons and addressed D3 function in their axons. Intense D3-immunoreactivity was observed in axon varicosities in the external zone of the rat median eminence and the neurohaemal zone of the human infundibulum containing axon terminals of hypophysiotropic parvocellular neurons. Immuno-electronmicroscopy localized D3 to dense-core vesicles in hypophysiotropic axon varicosities. N-STORM-superresolution-microscopy detected the active center containing C-terminus of D3 at the outer surface of these organelles. Double-labeling immunofluorescent confocal microscopy revealed that D3 is present in the majority of GnRH, CRH and GHRH axons but only in a minority of TRH axons, while absent from somatostatin-containing neurons. Bimolecular-Fluorescence-Complementation identified D3 homodimers, a prerequisite for D3 activity, in processes of GT1-7 cells. Furthermore, T3-inducible D3 catalytic activity was detected in the rat median eminence. Triple-labeling immunofluorescence and immuno-electronmicroscopy revealed the presence of MCT8 on the surface of the vast majority of all types of hypophysiotropic terminals. The presence of MCT8 was also demonstrated on the axon terminals in the neurohaemal zone of the human infundibulum. The unexpected role of hypophysiotropic axons in fine-tuned regulation of T3 availability in these cells via MCT8-mediated transport and D3-catalyzed inactivation may represent a novel regulatory core mechanism for metabolism, growth, stress and reproduction in rodents and humans.

## Introduction

Thyroid hormone is essential to normal brain development and function [1], [2]. Thyroxine (T4) is transported through the blood-brain barrier and converted to triiodothyronine (T3) to bind and activate thyroid hormone receptors (TR). This pathway is catalyzed by type 2 deiodinase (D2) in glial cells [3], [4], [5] from which T3 exits for uptake into TR-containing neurons to establish a transcriptional footprint [6]. However, regulation of thyroid hormone economy in the CNS also utilizes a second deiodinase, type 3 deiodinase (D3), that inactivates thyroid hormone in neurons [7], [8], [9], [10]. Hence, the interplay between D2 and D3 is a crucial mechanism to achieve temporally and spatially controlled regulation of thyroid hormone action, as has been described during hypoxia-induced brain hypothyroidism [6].

The hypothalamic hypophysiotropic neurosecretory system regulates metabolism, stress, growth and reproduction [11], [12] in a thyroid hormone-dependent manner. The negative feedback regulation of the hypophysiotropic thyrotropin-releasing hormone (TRH)-synthesizing neurons is well known to play a critical role to maintain peripheral thyroid hormone levels [12]. Local hypothalamic T3 regulation is also indispensible for reproductive function [13], [14]. Furthermore, thyroid hormone is necessary for ACTH and GH secretion from the anterior pituitary [15], [16], [17].

While hypophysiotropic neurons are located in different hypothalamic areas including the hypothalamic paraventricular nucleus (PVN), arcuate nucleus and medial preoptic area [18], hypothalamic D2 activity is predominantly confined to the mediobasal hypothalamus where tanycytes, a specialized glial cell-type lining the wall of the third ventricle have been shown to be the predominant D2 expressing cell-type [3], [4], [19]. Regulation of T3 generation of these cells impacts the function of hypophysiotropic neurons [6], [14], [20]. Since the cell bodies of most hypophysiotropic neurons are located some distance from tanycytes, it is currently unclear how tanycyte-derived T3 affects hypophysiotropic neurons. The hypothalamic median eminence represents a locus where D2-expressing tanycytes and hypophysiotropic axons could interact. Therefore in the present study, we determined whether tanycyte-generated T3 could be taken up and metabolized by axon terminals of hypophysiotropic neurons in the median eminence. Accordingly, we studied cellular and subcellular localization of D3 in the axon terminals of hypophysiotropic neurons and investigated whether monocarboxylate-transporter-8 (MCT8), the predominant neuronal T3 transporter [21], [22], is localized on these terminals.

We demonstrate that in the median eminence, D3 is present in subsets of GnRH-, GHRH- CRH and TRH containing axon terminals in a system specific level, and is subjected to trafficking in axonal dense core vesicles. MCT8 is expressed in the majority of these axons. We conclude that the axonal uptake and local degradation of T3 in the axonal compartment of hypophysiotropic neurons may be a novel pathway to regulate T3 concentrations in the hypothalamic median eminence.

## Results

### Distribution of D3 Protein in the Median Eminence of the Rat

**Figure 1:**
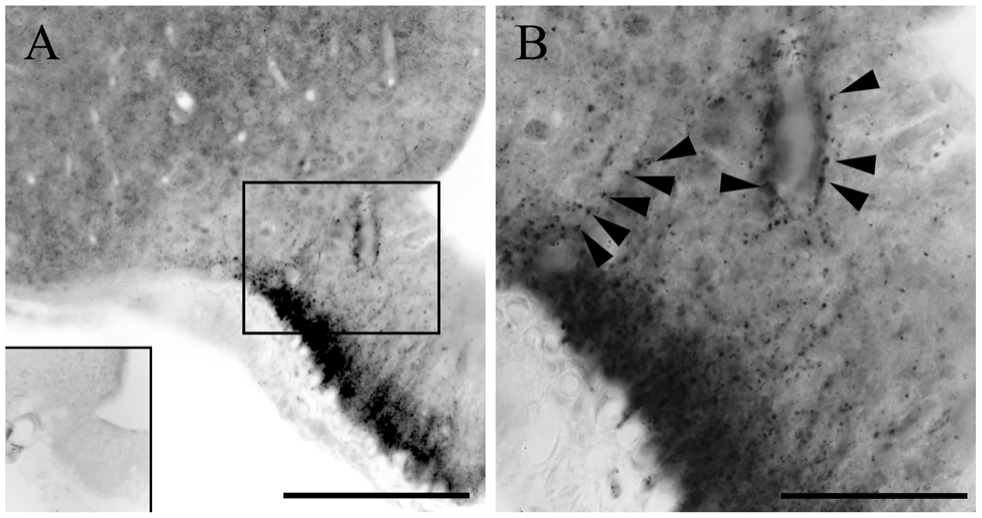
D3 immunoreactivity in the rat mediobasal hypothalamus. (**A**) Abundant D3 immunoreactive structures are seen in the external zone of the rat median eminence marked by silver grains. The inset on the left demonstrates the complete disappearance of D3- immunoractivity from the MBH when sections were incubated with D3 antisera previously preabsorbed with the corresponding peptide antigen. The boxed region is enlarged in (**B**). Black arrowheads indicate immunoreactive loci, which frequently were adjacent to blood vessels. Scale bars: 50 µm in A, 20 µm in B.

The D3-immunoreactivity appeared as small puncta distributed unevenly in the hypothalamus. The highest density was observed in the external zone of the median eminence (Fig. 1A–B), where the axons of the hypophysiotropic neurons accumulated around the portal capillary system. D3 immunoreactivity was also observed in most hypothalamic regions including those known to project to the median eminence (i.e. the medial preoptic area, paraventricular and arcuate nuclei), although less intense than the median eminence. The punctate appearance in these regions suggested localization in axons similar to that observed in axons in the median eminence as no D3 immunoreactivity was identified in neuronal perikarya.

**Figure 2:**
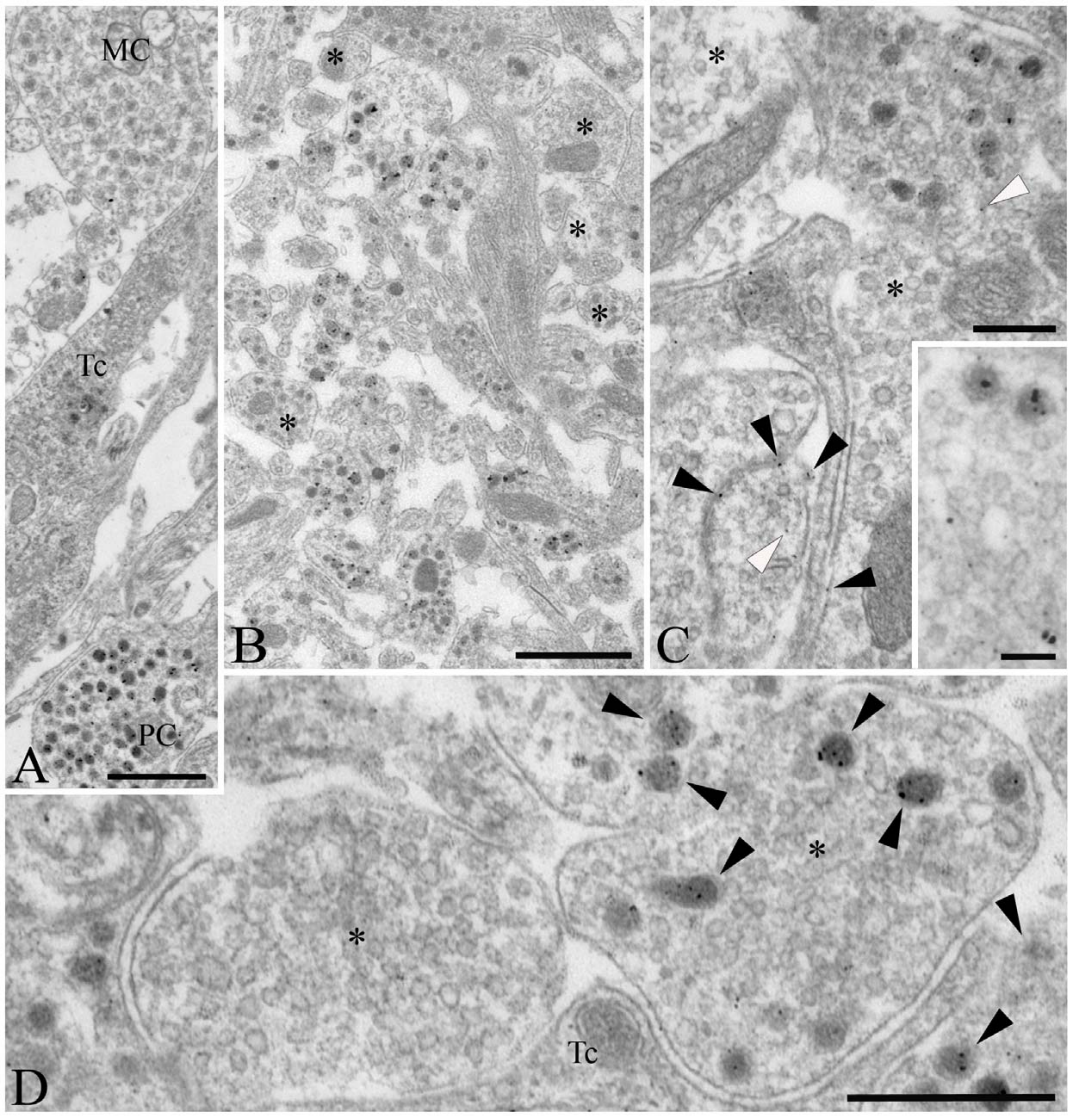
Ultrastructure of D3 immunoreactive elements in the rat mediobasal hypothalamus. (**A**) D3 immunoreactivity, identified by silver grain deposits, appear primarily in axon varicosities containing dense core vesicles of 80–120 nm diameter in the upper external zone of the median eminence, characteristic of axons of parvocellular (PC) neurons. No or a few silver grains could be observed in association with organelles of magnocellular neurons (MC) or tanycytes (Tc), respectively. (**B**) D3-positive axons exhibiting various degrees of labeling are mixed with non-labeled fibers (asterisks) in the external zone of the median eminence. (**C**) Although silver grains occasionally appear in association with the plasma membrane (black arrowheads) and with small clear vesicles (white arrowheads) of the axon varicosities, the majority are not labeled (asterisks). (**D**) In contrast, the dense core vesicles accumulate most the reaction product (black arrowheads), as visible at high power magnification in the vicinity of the capillaries of the external zone of the median eminence. Unlabeled small clear vesicles are indicated with asterisk. Tc, tanycyte; Scale bars: 1 µm in A–B, 250 nm in C, 100 nm in inset on C, 500 nm in D.

**Figure 3:**
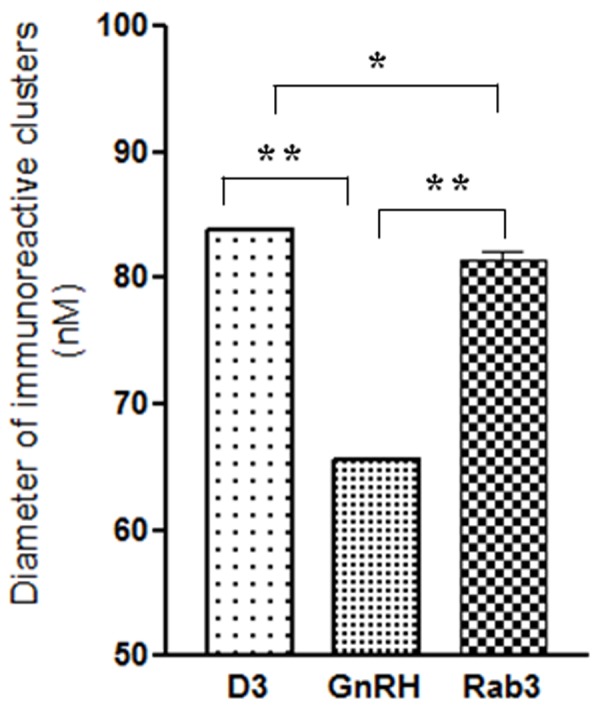
Diameter of immunoreactive clusters in the outer zone of the rat hypothalamic median eminence stained for D3, GnRH or Rab3 detected by N-STORM superresolution microscopy. **P<0.001; *P<0.05 by ANOVA followed by Newman-Keuls post test. Data are shown as Mean ± SEM (N = 500).

At the ultrastructural level, D3-immunoreactivity was localized exclusively to hypophysiotropic axon terminals in the external zone of the median eminence (Fig. 2). The majority of the silver grains denoting the D3-immunoreactivity were associated with dense core vesicles ranging between 80–120 nm (Fig. 2). Large dense core vesicles (200–350 nm), characteristic of magnocellular axons, were not labeled in the internal zone of the median eminence (Fig. 2A). A few scattered silver grains were also found in small clear vesicles and the plasma membrane (Fig. 2C). N-STORM superresolution microscopy was used to asses D3 topology in the dense core vesicles in the outer zone of the hypothalamic median eminence. The C-terminal portion of D3 formed immunoractive clusters of 83.9 nM, that was significanty larger than clusters containing intravesicular GnRH clusters of 65.6 nM and slightly bigger than clusters containing Rab3 (81.4 nM) (ANOVA followed by Newman-Keuls post-test, N = 500) a protein covering the outer surface of the dense core vesicles [23] (Fig. 3).

### Catalytic Activity and T3-mediated Regulation of the Axonal D3 Enzyme

**Figure 4:**
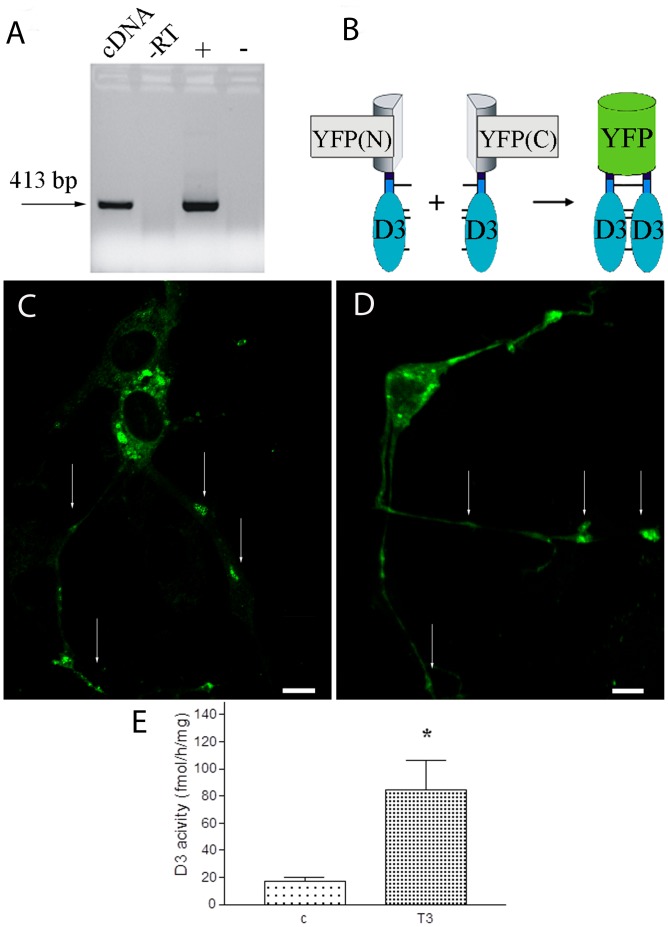
D3 expression in processes of mouse immortalized GT 1-7 GnRH neurons *in vitro.* (**A**) GT 1-7 cells endogenously express D3 mRNA. –RT: minus reverse transcriptase control; + and -: positive and negative controls, respectively (**B**) Schematic illustration of Bimolecular Fluorescence Complementation used to study D3 homodimerization. YFP(N) and YFP(C) stand for YFP(1-158aa) and YFP(159–238aa), respectively. (**C**) GT 1-7 cells were transfected with a plasmid encoding the fusion protein D3-YFP(full-length). (**D**) Co-expression of YFP(1-158)-D3 and YFP-(159–238)-D3 fusion proteins results in fluorescence complementation and demonstrates D3 homodimers in the axon-like processes of GT1-7 cells. Arrows indicate D3 along the processes. Scale bars: 10 uM. (**E**). Axonal D3 activity is increased by T3-treatment in the median eminence of male Wistar rats. Mean±SEM (n = 9) *P<0.05 by t-test.

To determine whether D3 could be catalytically active in axon terminals, we first determined whether homodimer formation, a feature required for D3 catalytic activity [24], occurs in the axon-like processes of the GT1-7 neurosecretory cell line that has both GnRH [25] and endogenous D3 expression (Fig. 4A). Bimolecular Fluorescence Complementation (BiFC) was used to detect dimerization between transiently expressed D3 monomers tagged with either the N or C-terminal fragment of YFP (Fig. 4B). First, the distribution of a transiently expressed D3 tagged with full-length YFP was studied. The D3-YFP fusion protein was observed in the axon-like processes of GT1-7 cells showing that a D3 monomer can be present in this compartment under the conditions used (Fig. 4C). To perform BiFC, the YFP-(1-158aa)-D3 and YFP-(159–238aa)-D3 were co-transfected and produced YFP activity in cell processes in a similar pattern as the monomers in Fig. 4C, confirming the presence of D3 homodimers in this compartment (Fig. 4D). No YFP signal was detected in negative controls using separate transfections of either the (YFP-(1-158aa)-D3 or YFP-(159–238aa)-D3 constructs, or in the absence of D3 after cotransfection of YFP-(1-158aa) and YFP-(159–238aa) (not shown). Importantly, D3 activity was detected in rat median eminence samples by deiodinase assay and this activity was up-regulated by ∼4-fold in hyperthyroid rats (17.3±2.8 *vs.* 84.5±21.4; mean±SEM, N = 3, p<0.05 by t-test) (Fig. 4E).

### Phenotype of D3- Immunoreactive Hypophysiotropic Terminals in the Rat Median Eminence

**Figure 5:**
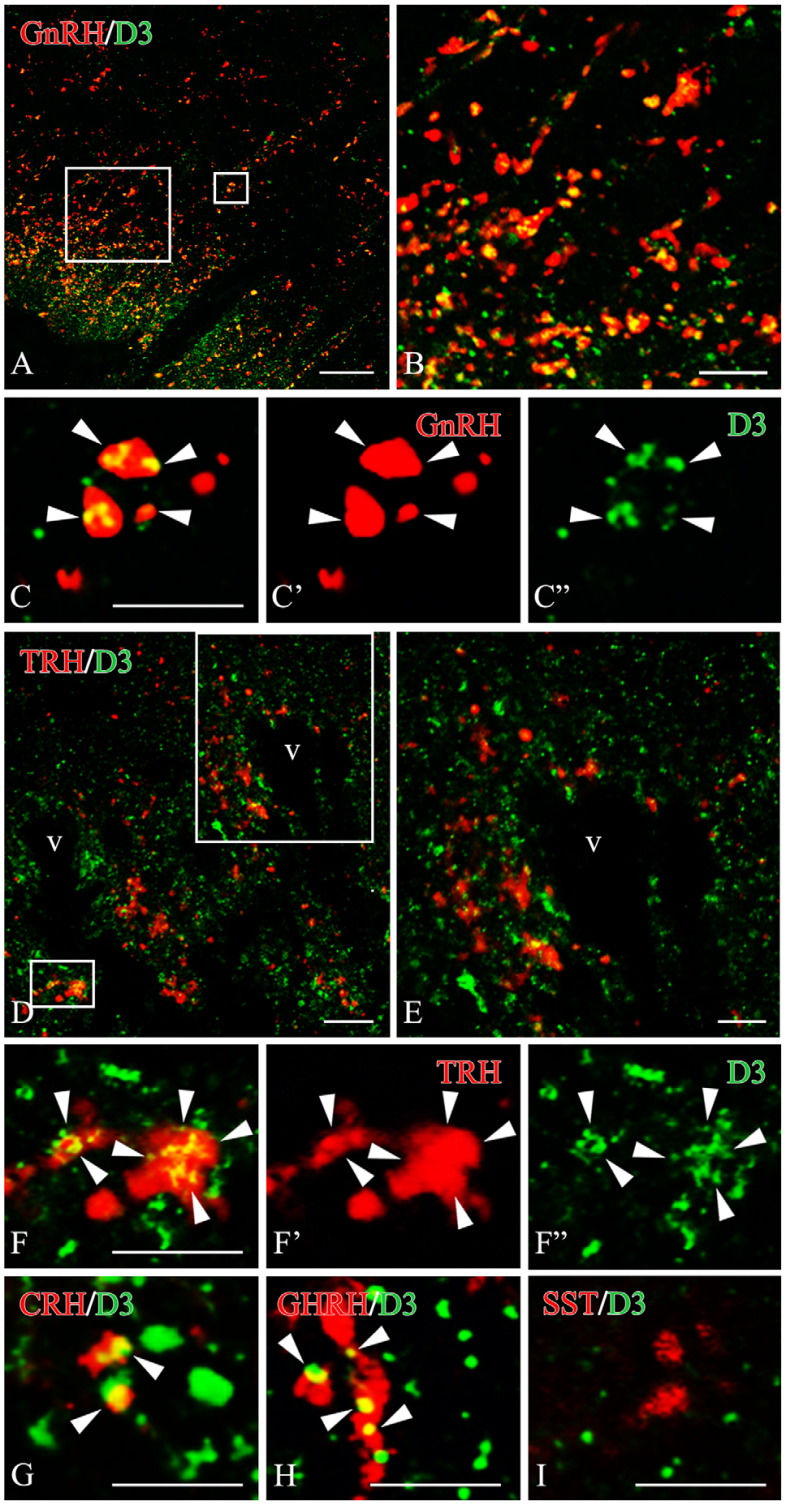
Dual-immunofluorescence images illustrate the overlapping distribution of fibers immunoreactive for D3 (green fluorochrome) and GnRH (A) or TRH (D) (red color) in the median eminence. Sites of overlap (yellow color) occur along the axonal pathway in the median eminence (**B,E**). High power confocal images demonstrate dual-labeled axon varicosities (yellow color in composite images) immunoreactive for D3 and GnRH (**C**) or D3 and TRH (**F**). The D3 immunoreactivity appears as yellow patches (arrowheads) within the axon varicosities. Single channels are also shown in **C’, C”** and **F’, F”**, respectively. High power dual-immunofluorescent images are also shown for fibers labeled for D3 (green fluorochrome) and CRH (**G**), GHRH (**H**) or somatostatin (**I**) (red color). These D3-immunoreactive sites (arrowheads) correspond to axon varicosities (**G**, **H**). Somatostatin (SS)- immunoreactive axon varicosities show virtually no signal for D3. Scale bars: 20 µm in A, 5 µm in B, 5 µm in C, 10 µm in D,E, 5 µm in F–I.

**Figure 6:**
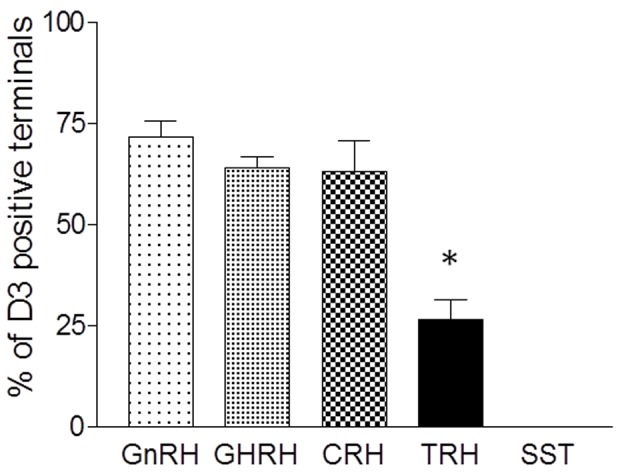
Quantification of D3 distribution in parvocellular axon terminals in the rat median eminence. (n = 3; *P<0.01 TRH *vs.* GnRH, GHRH, CRH and somatostatin (SST) by ANOVA followed by Newman-Keuls post-test.)

To determine the phenotype of the D3-containing axon terminals in the median eminence, co-localization of D3-immunoreactivity with hypophysiotropic releasing- or inhibiting hormones was performed with double-labeling immunofluorescence and confocal microscopy (Fig. 5). D3-immunoreactivity was observed in 71.8±3.8% of GnRH axon terminals (Fig. 5A–C). The D3-immunoreactive loci appeared as small islands within axon varicosities. In addition, D3 immunoreactivity was also detected in 63.2±7.5% of CRH- and 64.2±2.7% GHRH-immunoreactive axons, mostly in distal varicosities and terminal portions (Fig. 5D–F, and G, H). However, D3 was present only in 26.6±5.0% of TRH-immunoreactive varicosities. The lower D3 occurrence in TRH axons was significantly different from that observed in GnRH, CRH and GHRH axons (n = 3; *P<0.01 TRH *vs.* GnRH, GHRH, CRH by ANOVA followed by Newman-Keuls post-test) (Fig. 6). D3 was absent from somatostatin (SST)-immunoreactive axon varicosities (Fig. 5I, 6) and magnocellular neurons (not shown).

### MCT8-immunoreactivity is Present in the Hypophysiotropic Terminals of the Rat Median Eminence

**Figure 7:**
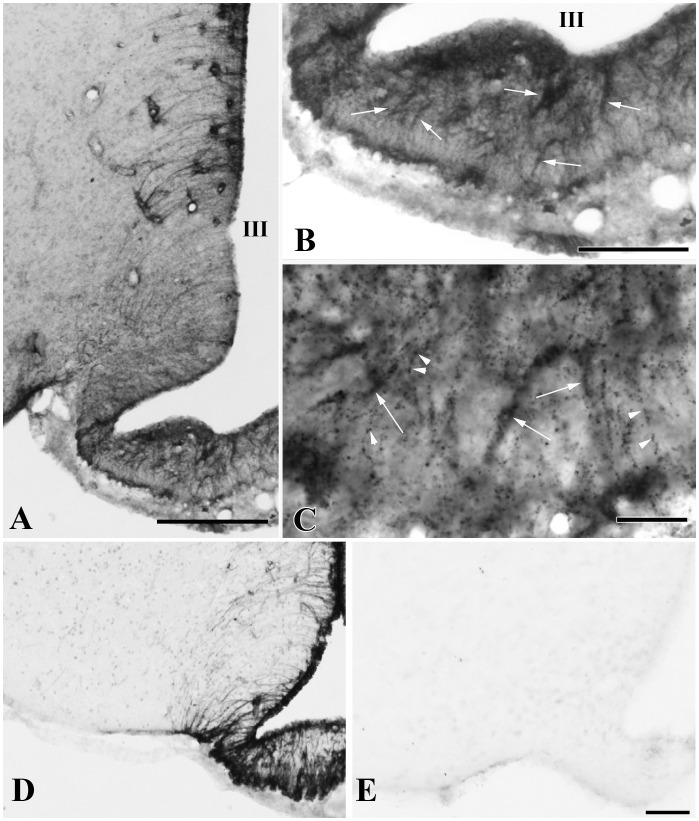
MCT8 immunoreactivity in the rodent mediobasal hypothalamus. Low magnification photograph illustrates the presence of MCT8-immunoreactivity associated with tanycytes (**A**). In the median eminence, strong MCT8-immunoreactivity is observed in tanycyte processes (**B, arrows**). In addition to occurring in tanycyte processes (arrows), MCT8-immunoreactivity is also observed in small dot like structures reminiscent of axon varicosities (arrow heads) (**C**). MCT8 immunoreactivity in the mediobasal hypothalamus of wild-type (**D**) and MCT8-KO mice (**E**). III, third ventricle; Scale bars: 200 µm in A, 100 µm in B, 20 µm in C, 50 µm in D,E.

**Figure 8:**
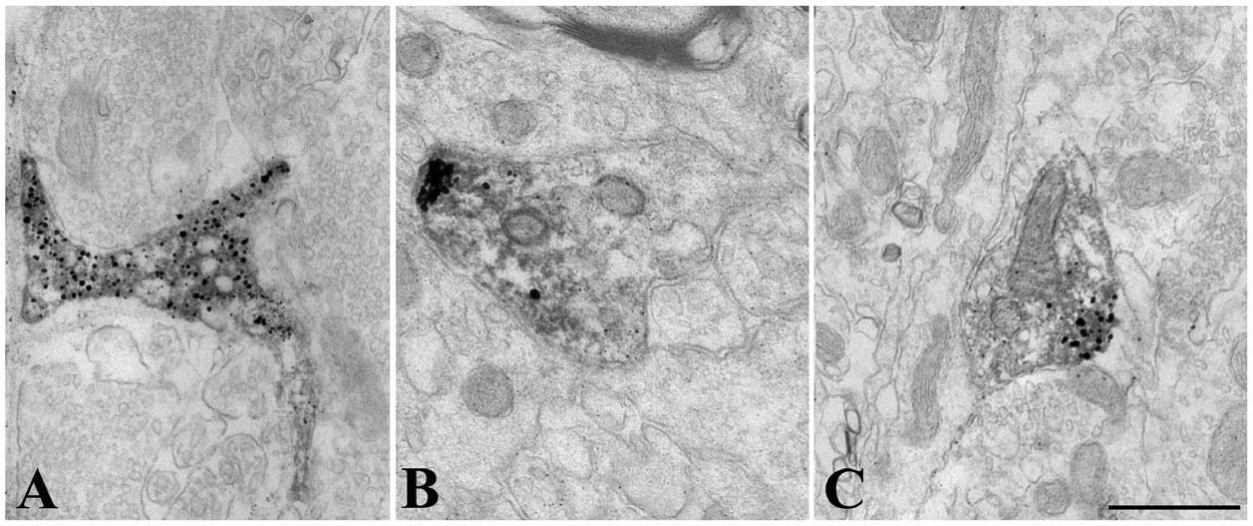
Ultrastructure of MCT8 immunoreactive structures in the rat median eminence. MCT8-immunoreactivity (silver grains) is associated with tanycyte (**A**) and axon varicosities (**B, C**) in the external zone of the median eminence. In the axon varicosities, silver grains accumulate in a small region of the varicosity close to the cytoplasmic membrane (**B, C**). Scale bar: 500 nM.

**Figure 9:**
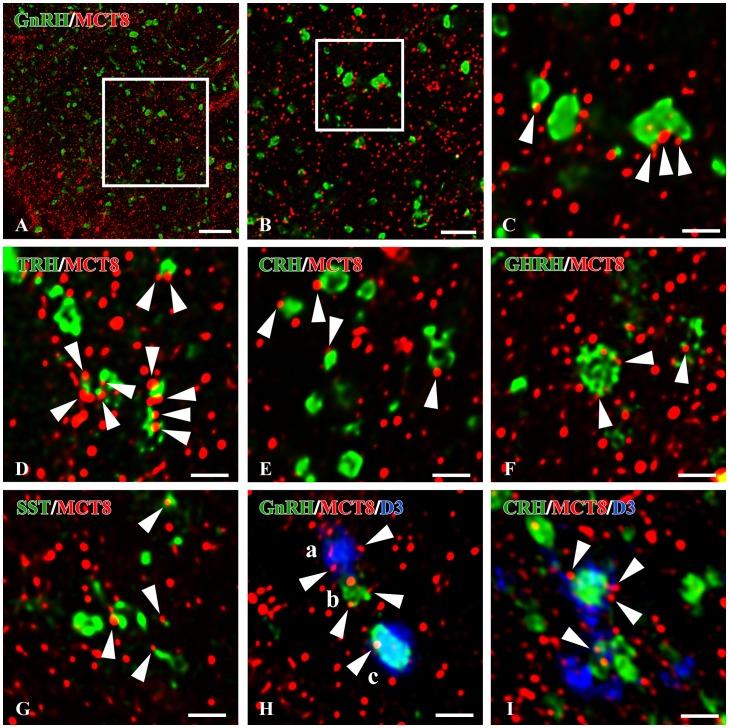
MCT8 and D3 immunoreactivities in axon varicosities of the rat parvocellular hypophysiotropic neurons. Confocal images were subjected to deconvolution. Boxed areas are enlarged in **B** and **C**, respectively. The immunofluorescent signal for MCT8 (red) is distributed as small dots throughout the external zone of median eminence and appears (arrowheads) on the surface of gonadotropin-releasing hormone (GnRH, **C**), thyrotropin-releasing hormone (TRH, **D**), corticotropin-releasing hormone (**E**), growth hormone-releasing hormone (GHRH, **F**) and somatostatin (SST, E) immunofluorescent axon varicosities (green). (**H,I**) Representative images of triple immunofluorescent labelings demonstrate D3 (blue), MCT8 (red) and a hypophyseotroph hormone (green) in the axons of the median eminence. MCT8-immunoreactive puncta (arrowheads) appear on the surface of the following categories of axon varicosities; single-labeled for D3 (**Ha**), single-labeled for GnRH (**Hb**), and double labeled for D3 and GnRH (**Hc**) or CRH (**I**). Scale bars: A: 10 µm, B: 5 µm, C-I: 2 µm.

To determine whether the D3-containing hypophysiotropic terminals are capable of accumulating T3, the distribution of MCT8-immunoreactivity was studied in the median eminence. Intense and diffuse MCT8-immunoreactivity was observed in cell bodies and processes exhibiting the characteristic distribution and morphology of tanycytes (Fig. 7A,B). In addition, punctate MCT8-immunoreactivity was detected among the tanycyte processes in the external zone of the median eminence (Fig. 7C). Ultrastructural analysis of the MCT8-immunoreactive elements in the external zone of the median eminence demonstrated strong MCT8-immunoreactivity distributed uniformly in the tanycyte processes (Fig. 8A). In addition, MCT8-immunoreactivity was also observed in axon terminals, where the silver grains focally accumulated in a segment of the axon varicosities in close proximity to the plasma membrane (Fig. 8B,C). A series of double-labeling immunofluorescent staining for MCT8 and hypophysiotropic peptides demonstrated the presence of MCT8-immunoreactive puncta on the surface of the vast majority of GnRH-, TRH-, CRH-, GHRH- and somatostatin-containing axon varicosities in the external zone of the median eminence (Figs. 9A–G).

### Colocalization of MCT8 and D3 in a Subpopulation of Hypophysiotropic Axons in the Median Eminence

In triple-labeled preparations, MCT8-immunoreactive puncta were observed on the surface of the vast majority of D3-immunoreactive axon varicosities containing either GnRH, CRH (Fig. 9H,I) and TRH, or GHRH (not shown). In contrast, somatostatin containing axons had only MCT8 without the presence of D3 (not shown).

### Distribution of D3 and MCT8 in the Infundibular Stalk of the Human Hypothalamus

**Figure 10:**
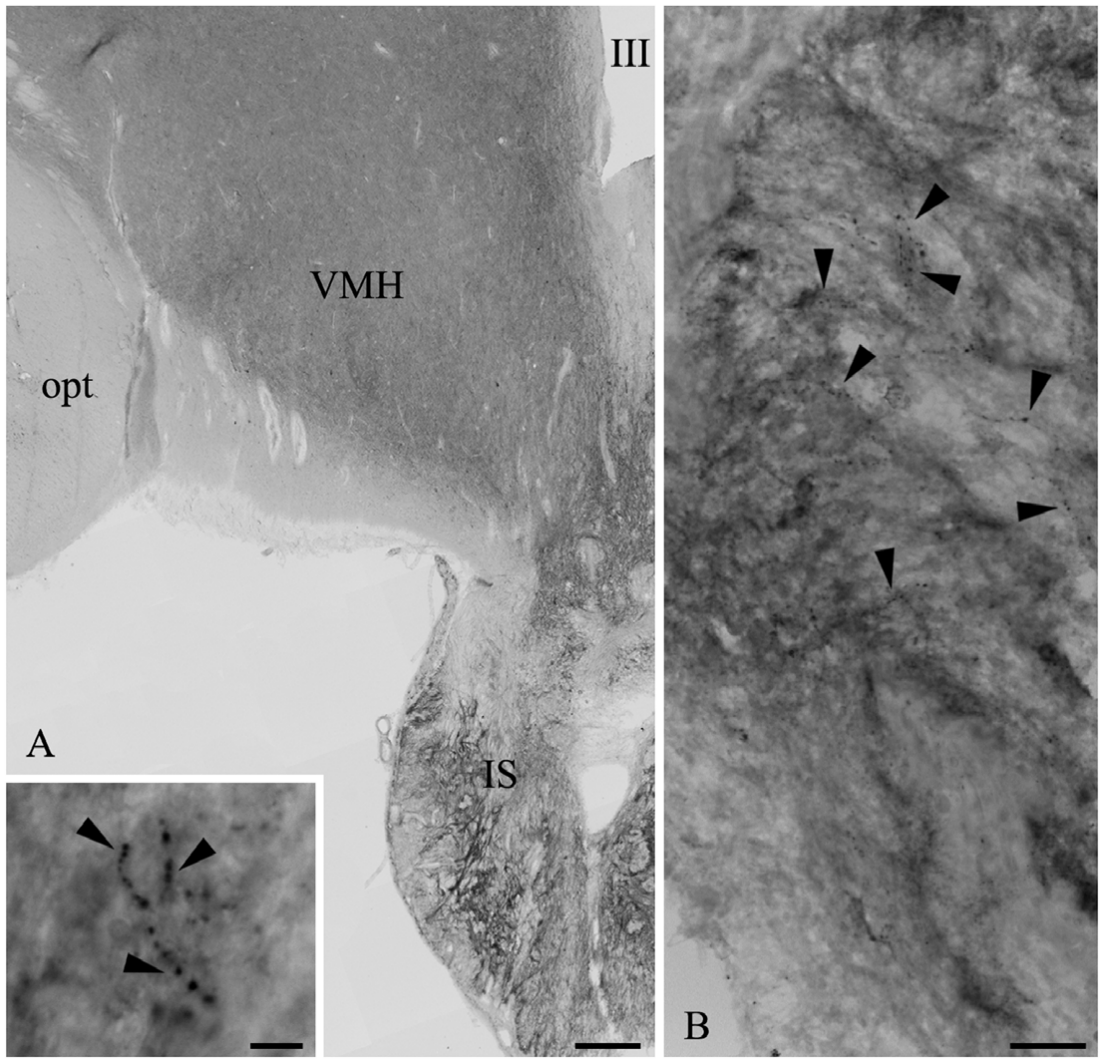
D3-immunoreactivity in the infundibular stalk of the human hypothalamus. (**A**) D3 immunoreactive fibers (arrowheads) are present in the human infundibular stalk (IS); these fibers are shown in medium (**B**) and high (inset in A) power micrographs. opt: tractus opticus, VMH: ventromedial hypothalamic nucleus, III: third ventricle Scale bars: 500 µm in A, 50 µm in B, 10 µm in inset.

**Figure 11:**
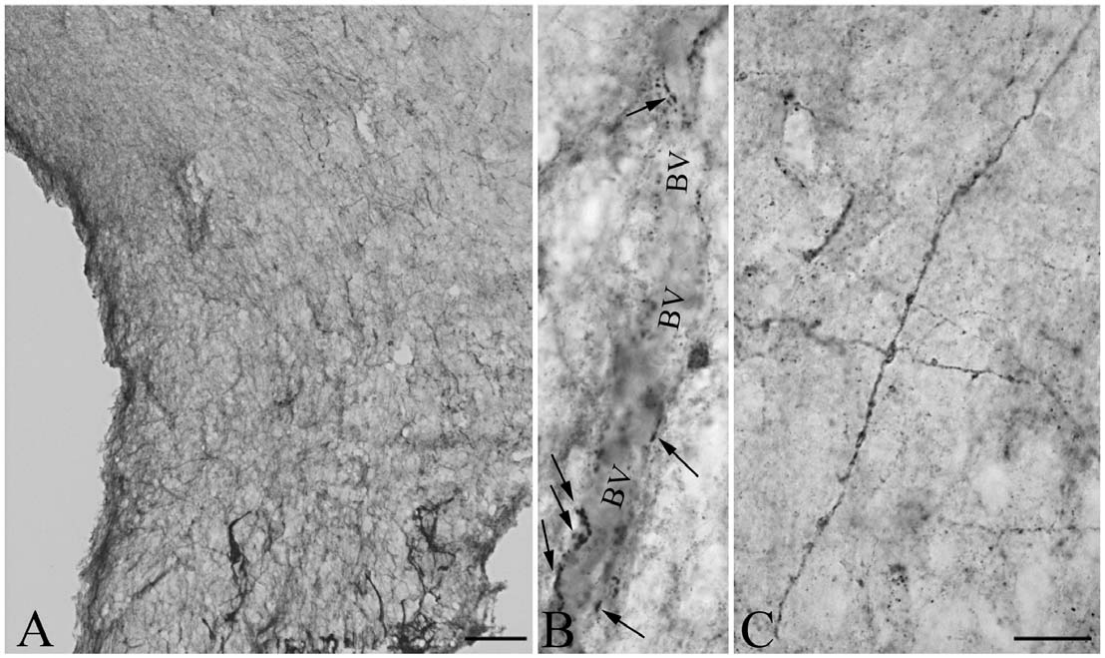
MCT8-immunoreactivity in the infundibular stalk of the human hypothalamus. A dense network of MCT8-immunoractive axons is present in the infundibular stalk (**A**)**.** A high magnification image illustrates MCT8-immunoractive axon varicosities in the proximity of a putative portal blood vessel (BV) in the neurovascular zone of the infundibular stalk (**B,** arrows)**.** Long, MCT8-immunoractive axons with large varicosities are frequently encountered (**C**). Scale bar: 100 µm in A, 20 µm in C (also corresponds to B).

Comparative studies in human mediobasal hypothalami showed a pattern of immunostaining for D3 and MCT8 similar to rat tissues. D3-immunoreactivity was present in the neurovascular zone of the human infundibular stalk where D3-immunoreactive puncta, reminiscent of axon varicosities, were detectable (Fig. 10A,B). MCT8 immunoreactivity was localized to various axons within the human infundibular stalk (Fig. 11A–C). The MCT8-immunoreactivity appeared in fibers exhibiting small varicosities (Fig. 11B) or relatively large swellings (Fig. 11C). MCT8 was also detected in tanycyte processes (not shown).

## Discussion

Although serum thyroid hormone concentrations are remarkably constant under physiological conditions, many biological processes require rapid and spatially controlled thyroid hormone action. Thyroid hormone transporters and deiodinase enzymes facilitate this purpose by enabling the trafficking of thyroid hormones and either increasing local thyroid hormone concentrations through the conversion of T4 to T3 by type 2 deiodinase, or degrading T3 by type 3 deiodinase [26], [27]. Collectively, the deiodinase enzymes are responsible for fine-tuned control of thyroid hormone levels and especially important in the brain to maintain thyroid hormone levels required for normal neuronal development and function [8]. In support of this concept is the observation that absence of D3 during neonatal development results in CNS abnormalities that are sustained into adulthood [28].

Thyroid hormone also plays a critical role in the regulation of hypothalamic function. Beyond its well-described role in regulation of hypophysiotropic TRH neurons, thyroid hormone has a complex impact on the regulation of other hypothalamic-pituitary axes [29], [30] including, the reproductive axis, adrenal axis and GH secretion [11], [13], [14], [15], [16], [17]. To further our understanding of how these neuronal systems are regulated by thyroid hormone, we identified the location and subcellular distribution of D3 and the major neuronal T3 transporter, MCT8, in hypophysiotropic neurons. These neurons have a common locus of termination in the external zone of the median eminence in close juxtapositon to the portal capillaries into which they secrete to modulate hormone production in the anterior pituitary. Their cell bodies of origin, however, are more widely distributed in the hypothalamus including the arcuate nucleus, paraventricular nucleus and preoptic region [31], [32].

D3-immunoreactivity was highly enriched in the external zone of the median eminence, and the authenticity of the signal was established by Western blot, showing a band identical that previously reported for D3 in the human [33]. In contrast to D2, which is located in tanycytes in the median eminence, D3 was seen in axon varicosities, primarily in dense core vesicles of neurosecretory granules of hypophysiotropic axons, while only limited immunostaining was also present in the plasma membrane of axon terminals. N-STORM superresolution microscopy revealed that the size of the D3-immunoreactive clusters had similar size as the RAB3-immunoreactive clusters and had significantly larger diameter than the GnRH-immunoreactive clusters. Since RAB3 is known to be located on the outer surface of the dense core vesicles and GnRH is packaged inside the dense core vesicles, this data indicate that similarly to RAB3, the C-terminal of D3, containing the peptide that was used for the generation of the antiserum, is also located on the outer surface of the dense core vesicles. As the C-terminal globular domain of D3 containing the active center of the enzyme is located in the cytosol, this localization allows an easy access to substrate. The transmembrane proteins with single transmembrane domain are classified according to their membrane orientation. Type 1 transmembrane proteins are single pass molecules with their C-terminus exposed to the cytosol [34]. Since D3 has one transmembrane domain on its N-terminal end [35], our findings provide *in vivo* evidence that D3 is a type 1 transmembrane protein and suggest that in hypophysiotropic neurons D3 exerts its biologic effects primarily in the membrane of dense core vesicles. Functional importance of D3 in axon terminals was proved by the demonstration of D3-homodimerization, a prerequisite for D3 catalytic activity [24], in cellular processes of GT1-7 cells and by the demonstration that elevated T3-levels evoke increased D3-medited axonal thyroid hormone in the rat median eminence.

Not all axons in the external zone of the median eminence were observed to contain D3 imunoreactivity. Rather, D3 was most prominently associated with GnRH-containing varicosities (71.8±3.8%). Thyroid hormone is known to have critical role in the regulation of the reproductive axis both in adult and developing animals [36]. Transient hypothyroidism during development has a major impact on the number and distribution of GnRH neurons in the hypothalamus [37]. Furthermore, thyroid hormone is essential for the photoperiod induced transition between the breeding phase and anestrus in seasonal breeding animals [38]. Since GnRH neurons express thyroid hormone receptors [39], the presence of D3 contained within GnRH terminals indicates that thyroid hormone may have an essential role in the regulation of reproductive function through direct effects on GnRH neurons. Thus, under certain conditions, controlling the amount of T3 within the GnRH neurons may be important to maintain normal function of the reproductive axis. This is supported by the phenotype of the D3 KO mouse in which deficits in reproductive function are observed [28]. Thyroid hormone also has a role in regulating the pulse frequency of GnRH in rhesus monkeys, particularly at the end of juvenile development when there is a thyroid hormone dependent resurgence in pulsatile GnRH release [36].

In addition to GnRH axon terminals, D3 was also prominently associated with CRH- and GHRH-containing axon terminals. Hypophysiotropic CRH neurons are well known to be regulated by thyroid hormone. Hypothyroidism decreases CRH gene expression in the PVN, while T4 replacement induces upregulation of CRH mRNA levels [40]. Furthermore, experimental hyperthyroidism results in a hyperexcitability of the hypothalamic-pituitary-adrenal axis [41]. Therefore, prevention of an increase in T3 concentration by axonal D3 may be beneficial for normal functioning of the adrenal axis. Relatively less is known about thyroid regulation of the GHRH neurons, although hypothyroidism results in increased GHRH synthesis and release [42], and the severe growth retardation of the D3 KO mice [28] suggests the importance of D3 in the regulation of the GHRH neurons. The presence of D3 in GHRH axon terminals raises the possibility that some of these effects may be exerted directly on the GHRH neurons.

At first glance, the relative paucity of D3 in axon terminals of hypophysiotropic TRH neurons (26.6%) might seem surprising, given that negative feedback effects of thyroid hormone on these neurons are so important for regulation of the hypothalamic-pituitary-thyroid axis [12]. The presence of D3 in neurons, however, may serve to modulate intracellular thyroid hormone levels, perhaps as a way to maintain constant thyroid hormone levels despite alterations in circulating levels. This type of regulation would not be to the advantage of hypophysiotropic TRH neurons that must sense increases or decreases in circulating levels of thyroid hormone to enhance or diminish anterior pituitary TSH secretion. Nevertheless, a small subpopulation of TRH-containing axon terminals did co-contain D3, indicating a heterogeneity among hypophysiotropic TRH axon terminals. A heterogeneity of the hypophysiotropic TRH neurons was also suggested by [43] showing that different subsets of hypophysiotropic TRH neurons respond to cold and suckling. Further studies are needed to understand whether the D3 expressing TRH neurons correspond to TRH neurons activated by cold environment, in which intracellular metabolism of T3 by D3 when circulating levels of thyroid hormone are elevated may be advantageous to promote upregulation of TRH gene expression.

In addition to the expression of D3 in axon terminals, the majority of axon varicosities in the median eminence express the T3 transporter, MCT8. This transporter is considered to be the predominant, neuronal T3 transporter, and mutations thereof in humans are characterized by a severe neurologic phenotype; [21], [22], [44]. As tanycytes express both MCT8 and OATP1C1 thyroid hormone transporters [33], [45], and type 2 deiodinase [4], these cells are capable of accumulating T4, converting T4 to T3, and then releasing T3 into the surrounding neuropil. As axon terminals in the external zone of the median eminence lie in close proximity to tanycyte endfeet processes [46], the observation that practically all hypophysiotropic axon terminals in the median eminence express MCT8 indicate that T3 could readily accumulate in the majority of hypopysiotropic axon terminals and then reach the nucleus of these cells by retrograde transport. Although the machinery driving the retrograde transport of T3 is yet unknown, fast retrograde axonal transport of biologically active molecules is not unprecedented [47]. Since the perikarya of the hypophysiotropic neurons are located relatively far from the tanycytes, it is likely that the retrograde axonal transport of T3 is the main route of T3 trafficking between tanycytes and hypophysiotropic perikarya.

T3 influx may also be directed to axonal mitochondria, affecting mitochondrial biogenesis and/or modulation of uncoupling proteins with important consequences on oxygen consumption, ATP generation and heat production [48], [49]. Modulation of neuronal energy homeostasis via mitochondrium-coupled mechanisms is known to affect neurotransmission, a process that is highly energy dependent [50]. In agreement with this, robust uncoupling protein-2 (UCP2) expression has been demonstrated in the neuronal processes of the hypothalamic-hypophysial system, paralleled with decreased mitochondrial respiration and elevated hypothalamic temperature compared to the non-UCP2 expressing thalamus [51]. This suggests that T3 mediated changes of mitochondrial function in hypothalamic axons could serve as potential regulator of neurotransmission.

In addition to the rodent median eminence, we also observed D3 and MCT8 to be present in the neurohemal zone of the human hypothalamus, corresponding to the rodent median eminence. These species similarities indicate that the proposed mechanisms of axonal T3 uptake and regulation *via* degradation are conserved across evolution and importantly, also function in humans.

Based on the MCT8 and D3 content in hypophysiotropic terminals, we propose that hypophysiotropic axons in the median eminence can be divided into two categories. The first are axon terminals that can uptake T3 and regulate the intracellular T3 concentration by axonal D3. This type of axon can be protected from sudden changes of T3 concentration by maintaining normal levels of cytoplasmic T3. The second type are axon terminals that accumulate T3 but unable to regulate intracellular T3. These axons may be very sensitive to changes in local T3 concentration that may be important for normal hypophysiotropic neuronal function.

**Figure 12:**
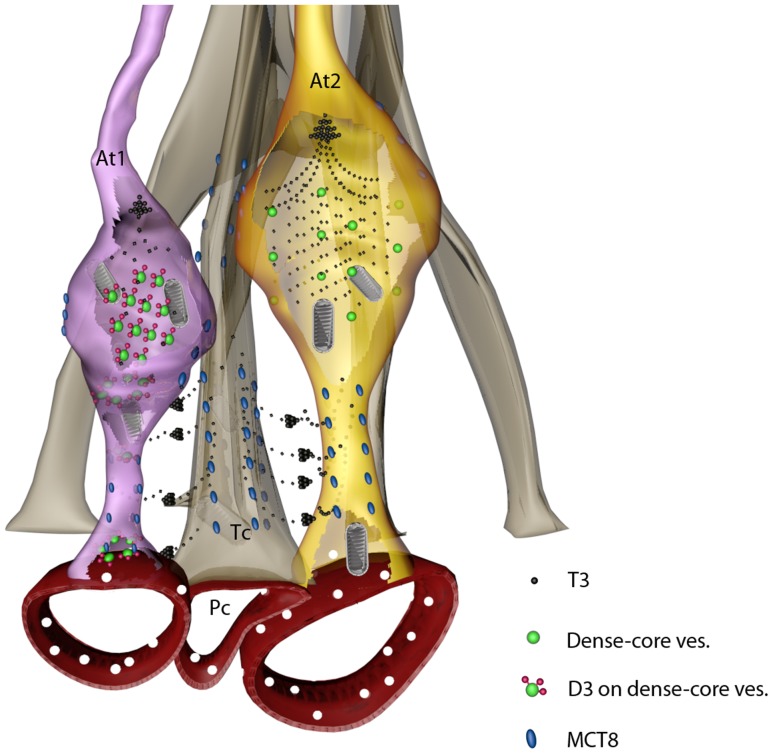
Schematic illustration of axonal uptake and regulation of T3 in the mediobasal hypothalamus. We suggest that T3 generated by D2 is released from tanycyte processes and taken up by MCT8 into axons of hypophysiotropic neurons. T3 concentrations are subjected to local regulation by D3-containing axon varicosities (At1), but absent in D3-negative axons (At2). T3 could be subjected to retrograde transport to reach the soma and nucleus of hypophysiotropic neurons and/or could act locally by affecting mitochondrial function and local thermogenesis. At, axon terminal; Tc, tanycyte; Pc, portal capillary.

In summary, we propose a novel concept of MCT8-mediated T3 uptake into two types of hypophysiotropic axons that either contain or lack D3 in the median eminence (Fig. 12). The strikingly different incidence of D3 in GnRH, GHRH, CRH and TRH or somatostatin neurons suggest a different capacity for T3 regulation in these neurosecretory cells. Axonal uptake and neuron-type specific regulation of intracellular T3 concentration in the rodent and human median eminence could represent a novel pathway for the modulation of hypothalamic control of reproduction, growth, stress and metabolism.

## Methods

### Animals

Adult, male Wistar rats (n = 20, b.w. 220–250 g, Toxi-Coop Ltd., Budapest) were kept under standard laboratory conditions with food and water *ad libitum*. Brains were perfused with a fixative solution described in the section of the specific experiments. Experimental procedures were approved by the Animal Welfare Committee of the Institute of Experimental Medicine and carried out in accordance with legal requirements of the European Community (Decree 86/609/EEC). Perfusion-fixed (4% PFA) brains of the MCT8-KO [52]. (n = 1) and the wild-type littermate mice (n = 1) were kindly provided by Dr. H. Heuer (Jena, Germany).

### Human Samples

For immunocytochemistry, human hypothalamic samples from two male and two female individuals who died from sudden causes of death were obtained at autopsy from the Forensic Medicine Department of the University of Debrecen, with the permission of the Regional Committee of Science and Research Ethics (DEOEC RKEB/IKEB: 3183-2010) according to Hungarian Law (1997 CLIV and 18/1998/XII.27. EÜM Decree/). All personal data were anonymized. Hypothalamic tissue blocks were dissected and immersion-fixed for 7–14 days with 4% PFA in PBS. For Western blot, fresh-frozen hypothalamic samples were obtained from the Human Brain Tissue Bank, Semmelweis University.

### Light Microscopic Immunohistochemistry for D3 and MCT8

Coronal 25–30 µm-thick sections through the anteroposterior extent of the median eminence of rats perfused with 4% paraformaldehyde in PBS (150 ml) and through the infundibulum of human samples were prepared on a freezing microtome (Leica Microsystems, Vienna, Austria) and processed for immunohistochemistry to study the distribution of D3- and MCT8-immunoreactivity. The sections were incubated in a mixture of 0.5% H_2_O_2_ and 0.5% Triton X-100 in PBS for 15 minutes to increase antibody penetration and reduce endogenous peroxidase activity. To reduce nonspecific antibody binding, the sections were treated with 2% normal horse serum in PBS for 20 minutes. To detect D3 immunoreactivity, sections of rat and human hypothalami were incubated (for 2 days at 4°C) in an affinity-purified, rabbit, polyclonal antiserum (0.5–1 µg/ml; NBP1-05767B; Novus Biologicals, Littleton. CO). MCT8 immunoreactivity was sought in hypothalamic sections of rats, humans and WT and MCT8-KO mice using a rabbit polyclonal antiserum (1∶5,000–10,000; kind gift of Dr. TJ Visser Rotterdam, The Netherlands). The primary antisera were reacted with biotinylated donkey anti-rabbit IgG (1∶500; Jackson ImmunoResearch, West Grove, PA) for 2 hours, followed by incubation in biotin-avidin-complex (ABC, 1∶1,000; Vector, Burlingame, CA) for 1 hour. The peroxidase signal was visualized with a NiDAB developer consisting of 0.05% diaminobenzidine, 0.15% nickel ammonium sulfate, and 0.005% H_2_O_2_ in 0.05 M Tris buffer (pH 7.6). The resulting reaction product was silver-gold-intensified using the Gallyas method [53], [54]. The immunostained sections were mounted onto glass slides from polyvinyl alcohol (Elvanol, Sigma, Budapest, Hungary), dried, and coverslipped with DPX mounting medium (Fluka, Buchs, Switzerland).

### Ultrastructural Detection of D3- and MCT8-immunoreactivities in the Rat Median Eminence

To study the cellular and subcellular distribution of D3 in the rat median eminence, 30–50 µm-thick coronal sections were cut from the 4% acrolein/2% PFA – fixed brains (n = 5) on a Vibratome. Excess aldehydes and endogenous peroxidase activity were eliminated by treatment in 1% sodium borohydride (30 min in PBS) and in 0.5% H_2_O_2_ (15 min in PBS), respectively. The sections were cryoprotected with 15% sucrose (15 min), then 30% (12 h) sucrose in PBS, followed by permeabilization using three sequential freeze-thaw cycles in liquid nitrogen. Finally, 2% normal horse serum was applied (20 min) to prevent nonspecific antibody binding. The pretreated sections were incubated in the primary antibodies (anti- D3 in 0.5–1 µg/ml or anti-MCT8 1:20,000 ) for 36–48 h at 4°C, followed by biotinylated donkey anti-rabbit IgG (1∶500) for 2 h and ABC complex (1∶1,000) for 1.5 h. The immunoreactive sites were visualized with NiDAB developer. Finally the immunoreaction product was silver-gold intensified [53], [54]. The sections were treated with 1% osmium tetroxide for 60 min and 2% uranyl acetate (prepared in 70% ethanol) for 40 min, dehydrated in an ascending series of ethanol and propylene oxide, and then, flat-embedded in TAAB 812 medium epoxy resin between a pair of glass microscope slides precoated with liquid release agent (Electron Microscopy Sciences, Hatfield, PA). The embedded sections were photographed and then, cut into ultrathin sections (50–60 nm) with a Leica Ultracut UCT ultramicrotome (Leica Microsystems, Wetzlar, Germany). The ultrathin sections were mounted onto Formvar-coated single slot grids, contrasted with 2% lead citrate and examined with a Jeol-100 C transmission electron microscope.

### Superresolution Microscopy (N-STORM)

Coronal 10 µm-thick sections through the anteroposterior extent of the median eminence of rats perfused with 4% paraformaldehyde in PBS (150 ml) were cut on a freezing microtome. The sections were pretreated for light microscopic immunocytochemistry as described above. The pretreated sections were incubated in one of the following antisera for 3 days at 4°C: rabbit anti-D3 serum (4 µg/ml), guinea pig anti-GnRH (#1018, 1∶4,000,000) [55], mouse monoclonal anti-Rab3a IgG clone 42.2 (1∶2000; Synaptic Systems). After washing in PBS, the sections were immersed overnight at 4°C in 1∶50 dilution of donkey anti rabbit, guinea pig or mouse IgG (Jackson Laboratories), respectively, doubly conjugated with CY3 (GE Healthcare) and Alexa 647 (Invitrogen). The Cy3/IgG ratio of the conjugated IgG was between 2 and 3, while the Alexa 647/IgG ratio was between 0.6 and 0.8. After washing in PBS, the sections were mounted on glass coverslip and air dried. Just before imaging the coverslipes holding the slides were mounted on glass slide using the following imaging medium: DPBS, 1 M mercaptoethylamine (MEA), 50% glucose solution in water, and the GLOX system (10 mg of glucose oxidase plus 25 ul of cata-lase and 100 ul of DPBS) in 80:10:10:1 volume ratio [56]. Axon varicosities located in the external zone of the median eminence were imaged using a Nikon N-STORM super-resolution microscope system (Nikon Instruments Ltd.) based on Nikon inverted Ti-E microscope equipped with perfect-focusing system (PFS) and the Nikon 100x NA 1.49 oil TIRF objective. A 561 nm wavelength laser (Sapphire 561-100-CW, Coherent) was used for excitation of the activator dye (Cy3) while a 647 nm wavelength laser (MPB Communications Inc, Montreal, Canada) was applied for excitation and bleaching of the reporter dye (Alexa647). The 2D images were acquired with an Andor Ixon DU-897 EMCCD camera (AndorTechnology,Belfast, Northern Ireland) using 30 ms exposition, one activation frame followed by three frames of imaging for 4000 cycles. The image trajectories were analysed by the N-STORM 2 module of the NIS-Elements followed by exporting as a high resolution bitmap (1.4 nm/pixel).

Using the NIKON NIS software, the diameter of at least 500 immunoreactive clusters from each staining was measured.

### Immunofluorescent Double-labeling for D3 and the Parvocellular Releasing- or Release-inhibiting Hormones

Immunofluorescent colocalization experiments were carried out on tissue sections fixed with 4% PFA (for detection of GnRH, CRH, GHRH and somatostatin) or 4% acrolein/2% PFA (for detection of TRH). Acrolein was inactivated with 1% sodium borohydride (30 min). All sections were pre-treated with H_2_O_2_ combined with Triton X-100 (0.5% each in 0.1 M PBS, 20 min), followed by normal horse serum (2% in 0.1 M PBS) for 10 min. First, the sections were incubated in rabbit anti-D3 antiserum (2 µg/ml, 48 h), which was detected with biotinylated-donkey anti-rabbit IgG (1∶500, 2 h) and Alexa-488-conjugated streptavidine (1∶400, 12 h). Then, one of the following primary antibodies (48 h, 4°C) were used: guinea pig anti-GnRH (#1018, 1∶5,000); sheep anti-TRH (#08W2, 1∶1,500) [57]; guinea pig anti-CRH (#T-5007, Bachem, 1∶3,000); sheep anti-GHRH (#19–4, 1∶30,000, kindly donated by Dr. I Merchenthaler Baltimore, MD, USA) [58]; rat anti-somatostatin (#354; Chemicon, 1∶50). These primary antibodies were reacted (12 h, 4°C) with appropriate CY3-conjugated secondary IgG raised in donkeys (1∶500, Jackson ImmunoResearch Laboratories, Inc.).

For quantification of the colocalization of D3 with the hypophysiotropic peptide, confocal images were taken from the external zone of the median eminence using 60 X oil immersion objectives. The fluorochromes were detected with the following laser lines and filters: 488 nm for Alexa 488 and 543 nm for Cy3 and dichroic/emission filters 560 nm/500–530 nm for Alexa 488 and 560–625 nm for Cy3. Pinhole sizes were set to obtain optical slices less than 0.7 µm thick. Colocalization of D3 and the other peptides were counted in the axon varicosities in the external zone. For each double labeling combination, brain sections from three animals were used. One randomly selected microscopic field (2600 µm^2^ each) from the external zone of median eminence of each animal was analyzed. Every field was divided into 100 equal parts to facilitate the counting of the double-labeled axon varicosities and the percentage of D3 occurrence in immunostained varicosities was determined.

### Immunofluorescent Double-labeling for MCT8 and the Parvocellular Releasing- or Release-inhibiting Hormones

Sections were pretreated identically as described above were incubated in rabbit anti-MCT8 antiserum (1∶1000, 48 h), and detected with Alexa 555-conjugated anti-rabbit IgG (1∶500, 2 h). Then, one of the following primary antibodies (48 h, 4°C) were used: guinea pig anti-GnRH (#1018, 1∶5,000) [55]; sheep anti-TRH (#08W2, 1∶1,500) [57]; guinea pig anti-CRH (#T-5007, Bachem, 1∶3,000); sheep anti-GHRH (#19–4, 1∶30,000, kindly donated by Dr. I Merchenthaler Baltimore, MD, USA) [58]; rat anti-somatostatin (#354; Chemicon, 1∶50). These primary antibodies were reacted (2 h) with appropriate FITC-conjugated secondary IgG that were raised in donkeys (1∶50, Jackson ImmunoResearch Laboratories, Inc.).

Confocal images were taken from the external zone of the median eminence using 60 X oil immersion objectives. The fluorochromes were detected with the following laser lines and filters: 488 nm for FITC and 543 nm for Cy3 and dichroic/emission filters 560 nm/500–530 nm for FITC and 560–625 nm for Cy3. Pinhole sizes were set to obtain optical slices less than 0.7 µm thick.

Labeled sections were scanned by using a Radiance 2100 confocal microscope (Bio-Rad Laboratories, Hemel Hempstead, UK). Deconvolution of 150 nm thick optical slices was performed using Xming (public domain at http://sourceforge.net/projects/xming/) software.

### Immunofluorescent Triple-labeling for MCT8, D3 and the Parvocellular Releasing- or Release-inhibiting Hormones

Half of the sections that were double-labeled for MCT8 and the parvocellular releasing- or release-inhibiting hormones were incubated in biotinylated rabbit anti-D3 antiserum (2 µg/ml, 48 h), followed by treatment in ABC (1∶1000, 2 h). The sections were then subjected to tyramide amplification according to the manufacturer’s instructions (NEN, Boston,MA). To further amplify the reaction product, the ABC treatment and the tyramide amplification were repeated. Finally the sections were incubated in CY5-conjugated streptavidin (1∶250).

Confocal images were taken from the external zone of the median eminence using 60 X oil immersion objectives. The fluorochromes were detected with the following laser lines and filters: 488 nm for FITC, 543 nm for CY3, and 637 nm for CY5; dichroic/emission filters, 560/500–530 nm for FITC, 650/565–625 nm for CY3, and a 660-nm-long pass filter for CY5. Pinhole sizes were set to obtain optical slices less than 0.7 µm thick. Deconvolution was performed as described above.

### Staining Specificity

**Figure 13:**
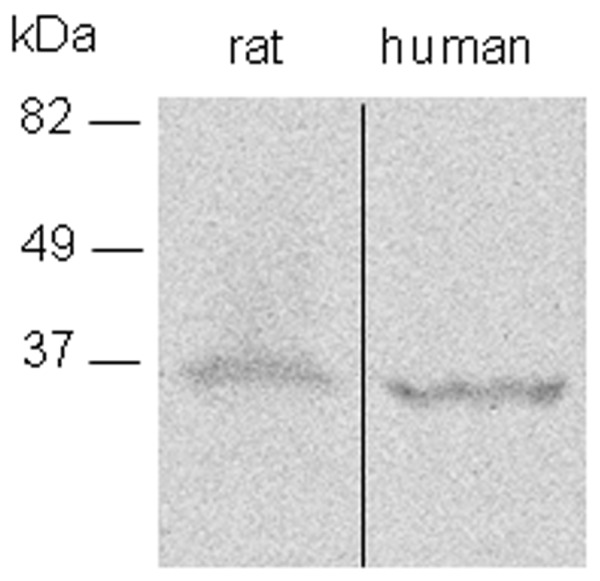
D3 detection by Western Blot in the rat and human hypothalamus. Note that the human D3 protein runs slightly faster compared to rat D3, in agreement with the calculated ∼3 kDa size difference between the two proteins.

The specificity of the D3 antiserum in the rat brain was described elsewhere [6]. To further determine the specificity of the antiserum in the examined region, the D3 antiserum was preabsorbed with the immunizing peptide (10 µg/ml) that resulted in loss of immunoreaction product in the MBH (Fig. 1A). The specificity of the antiserum was also demonstrated by Western Blot (see below) showing bands of expected size for rat and human D3 (Fig. 13). To test the specificity of the anti-MCT8 antibody in the examined region, hypothalamic sections of MCT8-KO mice were used as a negative control (the MCT8-KO brain was kindly provided by Dr. H. Heuer, Jena, Germany) [52]. MCT8-immunoreactivity was completely absent in the median eminence of the MCT8-KO mice (Fig. 7D,E). Specificity of the antisera against the hypophysiotropic hormones was earlier described [55], [57], [58]. The employed secondary antibodies were designed for multiple labeling and pre-absorbed by the producer with immunoglobulins of several species, including those in which the current non-corresponding primary antibodies were raised. Omission of any of the primary antisera from the triple-labeling immunofluorescence did not influence the pattern of the other two immunoreaction signals.

Western blot was performed using standard methodologies as described earlier [59]. Adult, male, Wistar rats were decapitated and the hypothalamus immediately frozen. A fresh-frozen human hypothalamic sample was obtained from the Human Brain Tissue Bank, Semmelweis University.

Fifty micrograms of rat and human hypothalamic protein sonicate was resolved by 10% SDS PAGE, and the D3 band identified using rabbit anti-D3 antibody (NBP1-05767, Novus, 0.5 ug/ul) and visualized using the BM Chemiluminescence Western Blotting Kit (Roche Diagnostics Co., Indianapolis, IN, USA). The blot showed bands of expected sizes of the rat and human D3 and also demonstrated the calculated 3 kDa size difference between the D3 proteins of the two species (Fig. 13.). After overexposure of the blot, faint bands of ∼22 and 120 kDa bands also appeared in the rat but not in the human sample.

### DNA Constructs

The constructs expressing human cysteine mutant D3 tagged with FLAG or YFP on its amino terminus have been previously described [24], [60]. For Bimolecular Fluorescence Complementation (BiFC), D3 was tagged at its N-terminus with YFP fragments. In short, the cys mutant D3 was generated with Vent polymerase, cut with BglII and BamHI and subcloned into the BglII site of the YFP-(1-158aa)pcDNAI and YFP-(159–238aa)pcDNAI vectors. The YFP BiFC fusion vectors were kindly provided by Dr. C Berlot (Danville, PA, USA) [61].

### Cell Culture, Transfection

The mouse GT 1-7 GnRH expressing cell line [25] was grown in DMEM-high glucose/F12 containing 10% FCS; 5% HS, supplemented with 3 mM glutamine. The D3-YFP mammalian expression constructs have been previously described [24]. Cells were transfected with Lipofectamine to transiently express the D3-YFP fusion proteins.

### Detection of Expressed D3 and Bimolecular Fluorescence Complementation (BiFC)

GT 1-7 cells were fixed with 4% PFA. The D3-YFP fusion protein was visualized directly with confocal microscopy. The YFP-(1-158aa)-D3 and YFP-(159–238aa)-D3 were coexpressed for (BiFC) and studied in 4% PFA fixed cells using confocal microscopy. The YFP-(1-158)-D3 and YFP-(159–238)-D3 constructs were also expressed separately while the YFP-(1-158aa) and YFP-(159–238aa) were coexpressed to serve as negative controls for BiFC.

### RT-PCR

RT-PCR was performed using standard procedures. In short, total RNA was isolated by Trizol (Invitrogen), reverse transcribed and amplified by Taq polymerase using mouse D3-specific oligonucleotides (sense, CCATATGCGTATCAGACGACAA; antisense, GTGCACCTTGTTGTAGTACTCT). Since the *Dio*3 gene is intronless, the RNA sample was also subjected to reverse transcription in the absence of reverse transcriptase enzyme (-RT control), then amplified by PCR to exclude the presence of genomic contamination.

### Animal Treatment and D3 Activity Assay

Adult male Wistar rats were injected *i.p.* with 50 µg/of T3/100 g body weight (N = 9) or vehicle (N = 9) every second days in 8 days. After decapitation the median eminence was dissected under a Zeiss Semi DV4 stereomicroscope (Carl Zeiss GMBH, Hamburg, Germany) and immediately frozen on dry ice. Three median eminence samples were pooled from nine, while five cortex samples were collected from five animals. The samples were sonicated in 0.1 M phosphate, 1 mM EDTA at pH 6.9 with 10 mM dithiothreitol and 0.25 M sucrose, and subjected to D3 assay as previously described [62].

### Statistics

All data are shown as mean ± SEM. Groups were compared with two-tailed t-test. Multiple comparisons were made by ANOVA followed by Newman-Keuls posthoc test.
